# CHRONIC PAIN CONTRIBUTES TO INJURIOUS FALLS IN COMMUNITY-DWELLING OLDER ADULTS

**DOI:** 10.1093/geroni/igz038.2318

**Published:** 2019-11-08

**Authors:** Yurun Cai, Suzanne Leveille, Ling Shi, Tongjian You, Ping Chen

**Affiliations:** University of Massachusetts Boston, Boston, Massachusetts, United States

## Abstract

Fall injuries are a leading cause of death among older adults, and chronic pain has been identified as a fall risk factor. However, the potential impact of chronic pain on injurious falls is unknown. This prospective study examined the relation between chronic pain and injurious falls in a 4-year follow-up of community-dwelling older adults. The MOBILIZE Boston study recruited 765 older adults aged ≥70y living in the Boston area. Pain characteristics, including pain severity, pain interference, and pain location, were measured at baseline using the Brief Pain Inventory subscales and a joint pain questionnaire. Musculoskeletal pain distribution was categorized as “no pain”, “single site pain”, or “multisite pain”. Injurious falls were ascertained in telephone interviews following reports of falls on the monthly fall calendar postcards. The overall rate of injurious falls was 35/100 person-years. Negative binomial models, adjusting for sociodemographics, BMI, chronic conditions, mobility difficulty, analgesic and psychiatric medications, and depression, showed that pain interference and pain distribution, but not pain severity, independently predicted injurious falls. Participants in the highest third of pain interference scores had a 53% greater risk of injurious falls compared to those in the lowest pain interference group (adj.IRR=1.53, 95% CI: 1.15, 2.05). Older adults with multisite pain had a 50% higher risk of injurious falls than those without pain (adj.IRR=1.50, 95% CI: 1.16, 1.93). Risk of injurious falls related to pain was stronger among women than men. Research is needed to determine effective strategies to prevent fall injuries among older adults with chronic pain.

